# Diagnostic Errors in Wilms' Tumors: Learning From Our Mistakes

**DOI:** 10.3389/fped.2021.757377

**Published:** 2021-10-25

**Authors:** Lucas Garschagen de Carvalho, Thiago Kobayashi, Monica dos Santos Cypriano, Eliana Maria Monteiro Caran, Henrique Manoel Lederman, Maria Teresa de Seixas Alves, Simone de Campos Vieira Abib

**Affiliations:** ^1^Pediatric Oncology Institute, GRAACC Hospital, Federal University of São Paulo, São Paulo, Brazil; ^2^Paulista School of Medicine, Federal University of São Paulo, São Paulo, Brazil

**Keywords:** Wilms, neuroblastoma, surgery, diagnostic errors, preoperative chemotherapy

## Abstract

**Aim:** This study aimed to analyze clinical characteristics and image findings in patients initially diagnosed with renal masses and treated on the Société Internationale d'Oncologie Pédiatrique (SIOP) 2001 protocol for Wilms tumor (WT) that eventually were diagnosed with different pathologies.

**Methods:** We reviewed the preoperative symptoms, laboratory tests, and images of patients who were initially treated for WT and proved to have other diagnoses. Data from these patients were compared to those of the last 10 patients with WT and the last 10 patients with neuroblastoma (NBL) treated at a single institution.

**Results:** From June 2001 to December 2020, we treated 299 patients with NBL and 194 with WT. Five patients treated with preoperative chemotherapy for WT were postoperatively diagnosed with NBL (one patient had bilateral renal masses and one with multifocal xanthogranulomatous pyelonephritis). Three underwent nephrectomy, two biopsies only, and one adrenalectomy due to intraoperative characteristics. Regarding clinical presentation, abdominal mass or swelling was very suggestive of WT (*p* = 0.011); pain, although very prevalent in the study group (67%), was not statistically significant, as well as intratumoral calcifications on computed tomography (CT) (67%). Urinary catecholamines were elevated in all patients mistreated for WT with the exception of the patient with pyelonephritis in which it was not collected.

**Conclusion:** Some pathologies can be misdiagnosed as WT, especially when they present unspecified symptoms and dubious images. Diagnostic accuracy was 98.1%, which highlights the quality of the multidisciplinary team. Abdominal mass or swelling is highly suggestive of WT, especially in the absence of intratumoral calcifications on CT. If possible, urinary catecholamines should be collected at presentation as they help in the differential diagnosis of NBL.

## Introduction

Wilms tumors (WT) and neuroblastomas are the most common diagnoses in children with palpable abdominal masses (1). Neuroblastoma is the fourth most common neoplasm in childhood, behind leukemias, brain tumors, and lymphomas, and is the most common extracranial solid tumor in children (2). It has the peak of incidence in the first year of life, and it is extremely rare after 5 years of age (3). Neuroblastomas originate from primitive ganglion cells from the neural crest that undergo transformation and migrate ventrally and caudally to form many tissues such as the branchial arches, thoracic vessels, sympathetic nervous system, and adrenal medulla. Due to this migration of neuroblasts in the embryonic period, neuroblastomas can be located in the abdomen, chest, neck, and pelvis and very rarely as an intrarenal tumor (4). On computed tomography (CT), the tumor typically is heterogeneous with calcifications seen in 80–90% of cases (5). Areas of necrosis are of low attenuation. The tumor morphology is often helpful, with the mass seen insinuating itself beneath the aorta and lifting it off the vertebral column. It tends to encase vessels and may lead to compression. Adjacent organs are usually displaced; although in more aggressive tumors, direct invasion of the psoas muscle or kidney can be seen. The latter can make distinguishing neuroblastoma from WT difficult.

WT is the most common renal neoplasm in children under 15 years of age and represents 95% of cases of renal masses in children. The peak incidence is between 2 and 4 years of age (6), and 95% of the cases are diagnosed before 5 years of age (7). Both neuroblastoma and WT can present with asymptomatic abdominal mass or pain and hypertension. WT can also present with hematuria (7). The diagnosis of neuroblastomas and WT is based on clinical history, physical examination, and laboratory tests and image. In suspected cases of neuroblastomas, urinary catecholamine should be collected (8), associated with a bone marrow aspirate and a primary tumor biopsy. Imaging exams assess the characteristics of the mass and the extent of the disease (9).

Ultrasonography is the main initial examination for the investigation of abdominal mass, allowing the identification of calcifications and tumor characteristics (9). In patients with suspected renal tumor, Doppler ultrasonography may demonstrate tumor extension to the renal vein and inferior vena cava. CT scan and magnetic resonance imaging (MRI) are performed to investigate the extension of the mass and its relationship with adjacent tissues (10).

The differential diagnosis between neuroblastoma, WT, and other kidney tumors is based on the patient's age, symptoms, and laboratory and imaging characteristics. In some cases, aspects of imaging studies are common among tumors, leading to a difficulty in preoperative diagnosis (8). There are some reports in the literature of xanthogranulomatous pyelonephritis presenting as WT (11).

Unlike the Children's Oncology Group (COG), which advocates the initial surgical approach and only after the diagnosis is confirmed is chemotherapy started, Société Internationale d'Oncologie Pédiatrique (SIOP) advocates preoperative (neoadjuvant) chemotherapy for WT, without an anatomopathological substrate, using only classic imaging findings (12) with a global diagnostic accuracy of 86% (13). When a case of neuroblastoma or non-Wilms kidney tumor is misdiagnosed by imaging studies as a WT, patients are treated with neoadjuvant chemotherapy not specific for the true histological type of the tumor, which can delay treatment and change the prognosis of the disease.

The aim of this study is to evaluate patients who received preoperative chemotherapy for WT and had different diagnosis.

## Materials and Methods

This is a retrospective study of children misdiagnosed with WTs, treated at the Pediatric Oncology Institute—GRAACC—Federal University of São Paulo, Brazil, from June 2001 to December 2020. The study was approved by the institutional review board (CEP #0808/13). All patients treated with preoperative chemotherapy for WT on the SIOP 2001 or SIOP 2016 (Umbrella) protocol whose pathology revealed a different diagnosis were included. Patients who had surgery as a first approach or were treated on other protocols were excluded. Initial symptoms, physical examination findings, laboratory tests, and imaging studies were retrospectively reviewed and compared to those of the last 10 patients treated for WT and neuroblastoma in the institution.

We use the main features of our imaging exam reports to perform data analysis. On ultrasound were heterogenicity, lobed outline, size, restriction of the lesion to the kidney, Doppler flowmetry, and laterality. In CT scan were heterogenicity, calcification, lymph node enlargement, capsule invasion, and size.

Statistical analysis was performed using the Fisher test and Student's *t*-test. In this study, an association was considered significant if the corresponding *p*-value was <0.05.

## Results

From June 2001 to December 2020, 299 cases of NBL and 194 of WT were treated at the Pediatric Oncology Institute—GRAACC—Federal University of São Paulo. Six patients received preoperative chemotherapy according to the SIOP protocol, and pathology proved to have other diagnoses. Five patients presented undifferentiated neuroblastoma as histopathological findings and one as xanthogranulomatous multifocal pyelonephritis. Diagnostic accuracy was of 98.1%.

**Figure 1:**
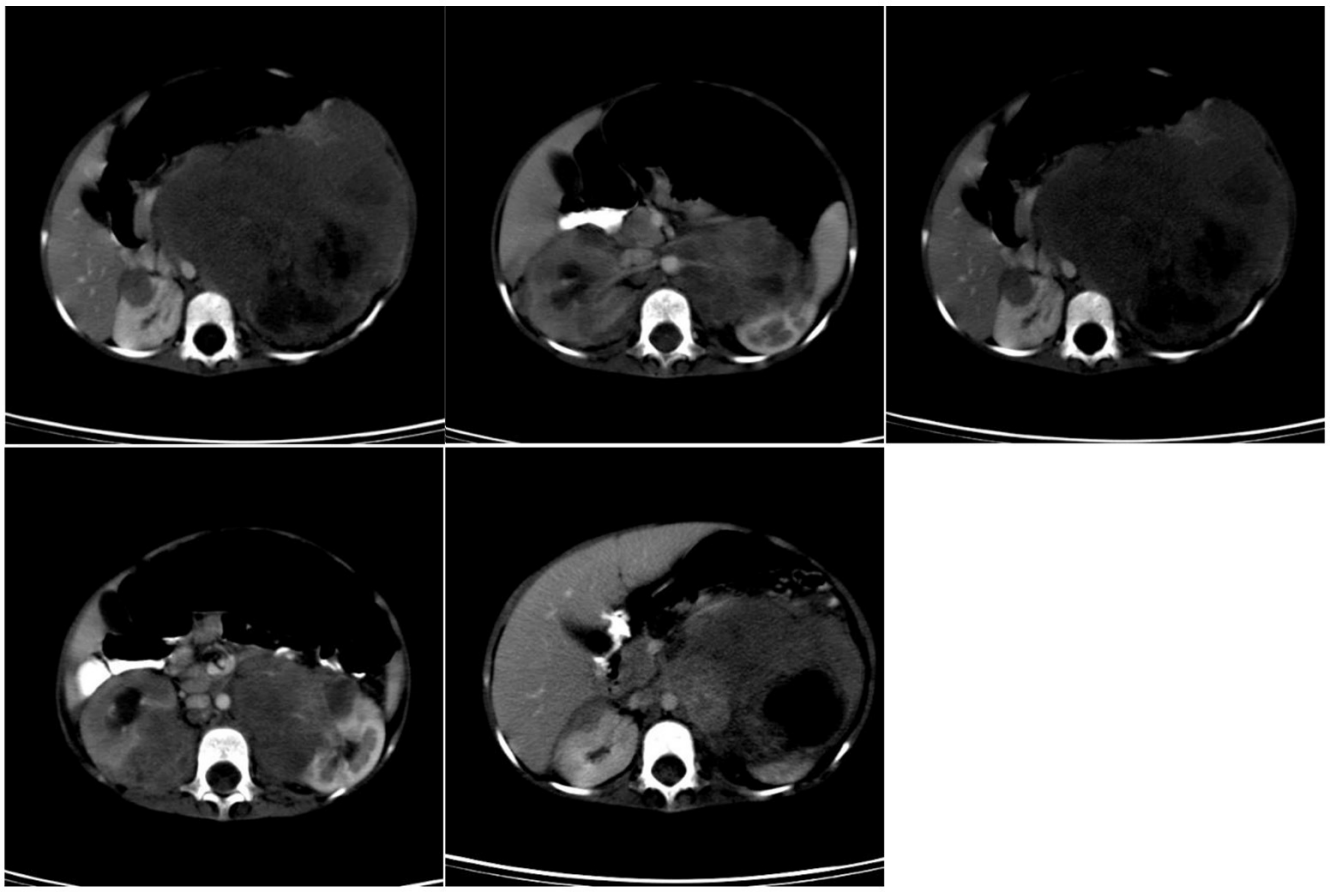
Preoperative CT scan of patient B.

**Figure 2:**
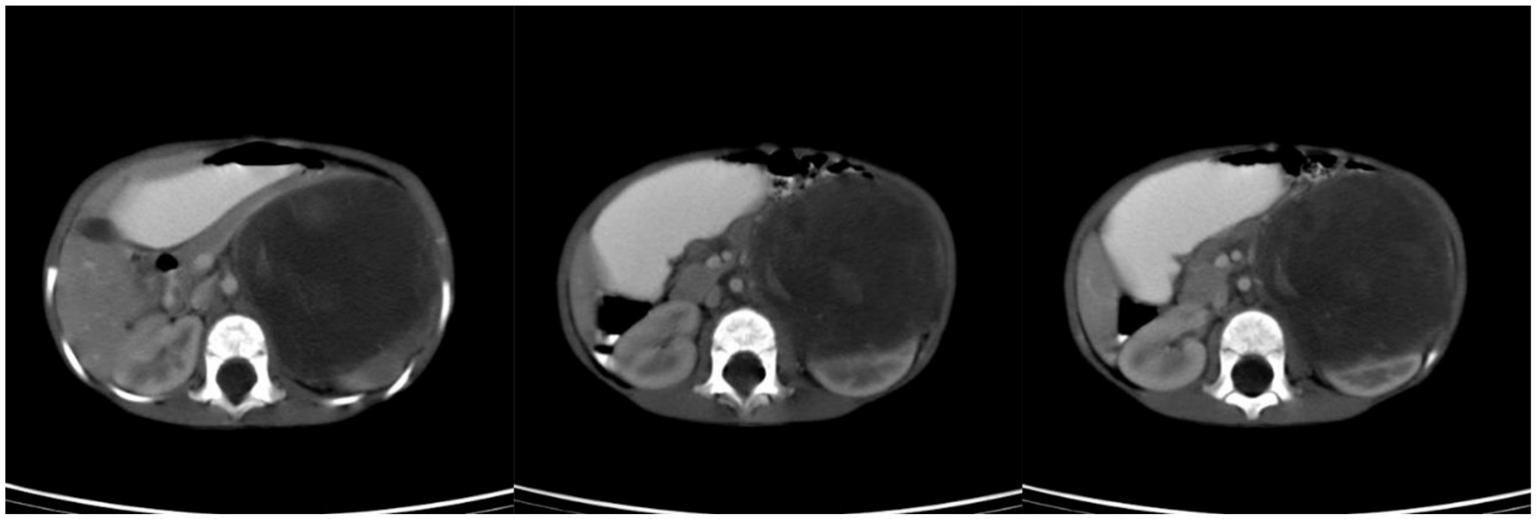
Preoperative CT scan of patient C.

**Figure 3:**
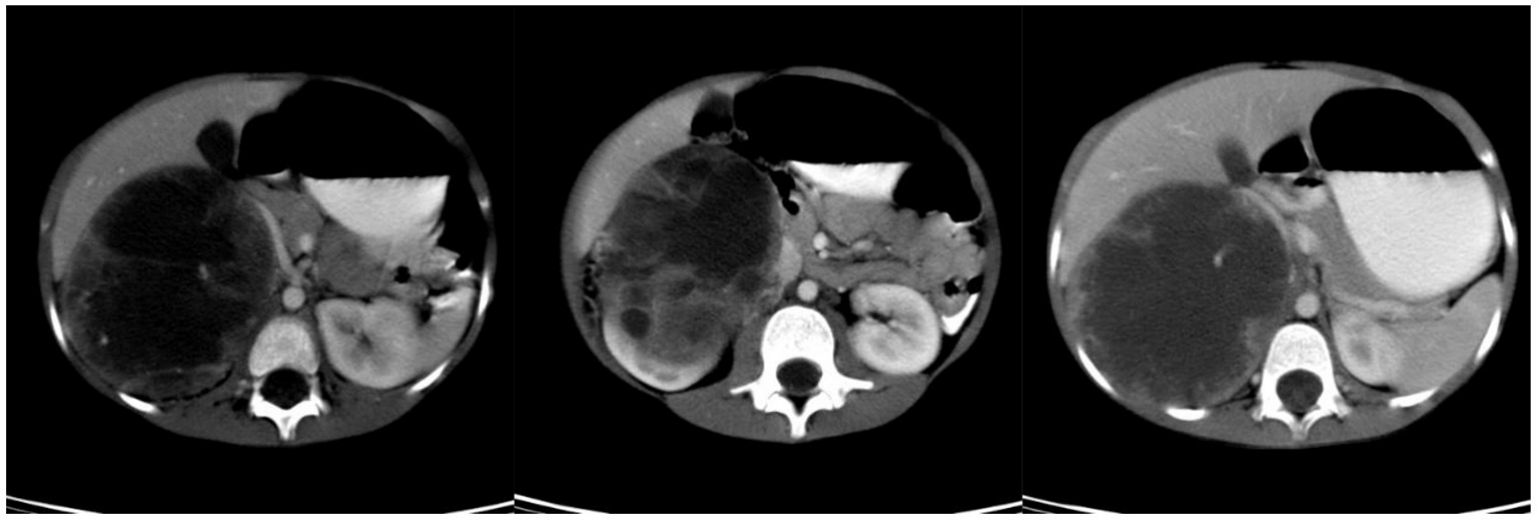
Preoperative CT scan of patient E.

Patient A after chemotherapy showed tumor growth with areas of necrosis. He underwent nephrectomy, adrenalectomy, splenectomy, and colectomy due to tumor invasion and died 2 years after surgery due to tumor progression despite the institution of neuroblastoma-directed therapy after the histological result. Patient B (Figure 1) had a bilateral tumor that showed a small reduction after chemotherapy. After two cycles of actinomycin and vincristine and one cycle of vincristine, biopsy was performed; due to the appearance of the tumor and due to the advanced stage of the disease, he was considered irressecable and died due to disease progression despite chemotherapy treatment. Patient C (Figure 2) presented tumor regression with chemotherapy, undergoing nephrectomy and adrenalectomy, and is still undergoing treatment. Patient D showed no response to chemotherapy and underwent nephrectomy and adrenalectomy. He is currently off therapy for 14 years. Patient E (Figure 3) presented tumor growth with necrosis after chemotherapy and underwent only tumor biopsy and died 1 month after surgery because of disease progression. Patient F had Beckwith–Wiedemann syndrome and had a 50% tumor reduction with chemotherapy. Right nephrectomy was performed, but histopathological findings confirmed a xanthogranulomatous multifocal pyelonephritis.

The identified patients showed smaller abdominal volume, when compared to the 10 WT patient controls (*p* = 0.011). In relation to the neuroblastoma controls, they presented greater increase in abdominal volume and less pain evaluated by the FLACC scale, however without statistical significance. The study group had higher levels of pain, when compared to the WT controls (67% vs. 20%, *p* = 0.0611), and numbers very similar to the neuroblastoma controls (67% vs. 70%, *p* = 0.889). None of the symptoms, such as fever, vomiting, diarrhea, weight loss, constipation or enterorrhagia, were statistically significant. No patient in the study group presented with hematuria as in the WT controls or with lymphadenomegaly and tremors as in the neuroblastoma controls (Table 1).

**Table 1:** Comparison between the study group, the Wilms group, and the neuroblastoma group in relation to symptoms.

	**Number of patients**	***p-*** **value**
**Increase abdominal volume**		
Study group	2	
Wilms group	10	0.011^*^
Neuroblastoma group	1	0.247
**Pain**		
Study group	4	
Wilms group	2	0.061
Neuroblastoma group	7	0.889
**Fever**		
Study group	2	
Wilms group	1	0.247
Neuroblastoma group	1	0.247
**Vomiting**		
Study group	2	
Wilms group	2	0.55
Neuroblastoma group	4	0.789
**Diarrhea**		
Study group	0	
Wilms group	1	0.606
Neuroblastoma group	2	0.43
**Weight loss**		
Study group	0	
Wilms group	1	0.182
Neuroblastoma group	1	0.606
**Constipation**		
Study group	1	
Wilms group	1	0.696
Neuroblastoma group	3	0.55
**Enterorrhagia**		
Study group	1	
Wilms group	0	0.282
Neuroblastoma group	0	0.282
**Hematuria**		
Study group	0	
Wilms group	1	0.606
Neuroblastoma group	0	–
**Adrenomegaly**		
Study group	0	
Wilms group	0	–
Neuroblastoma group	1	0.606
**Tremor**		
Study group	0	
Wilms group	0	–
Neuroblastoma group	1	0.606

* *Bold values means statistical significance (p < 0.05)*.

Comparing the study group to WT and neuroblastoma controls showed similar results regarding hemoglobin, leukocytes, and platelets. The LDH cannot be compared to the WT group due to lack of data; only three patients had these data available. In the neuroblastoma controls, this value was slightly lower than that in the study group and without statistical significance (716 vs. 627, *p* = 0.082). With the exception of patient A, homovanillic acid (HVA) and vanillylmandelic acid (VMA) were measured in all others in the study group. Although HVA was increased in all patients in the study group (58.8 vs. 239, *p* = 0.086) and VMA was normal only in patient E (29.2 vs. 82.3, *p* = 0.073), the values were below those of the neuroblastoma group and did not show statistical significance (Table 2).

**Table 2:** Comparison between the study group, the Wilms group, and the neuroblastoma group in relation to laboratory tests.

		***p-*** **value**
**Hemoglobin (g/dl)**		
Study group	10.7 (7.8–15.4)	
Wilms group	10.6 (8–13.5)	0.15
Neuroblastoma group	11.5 (8.9–14.4)	0.203
**Leukocytes (10^3^/μl)**		
Study group	10.4 (5.1–17.4)	
Wilms group	11.8 (7.1–21.2)	0.297
Neuroblastoma group	9.4 (5.1–17.0)	0.134
**Platelets (platelets/μl)**		
Study group	459,317 (21,800–696,500)	
Wilms group	414,350 (170,000–630,000)	0.55
Neuroblastoma group	346,230 (62,000–710,000)	0.894
**LDH (UI/L)**		
Study group	716 (282–950)	
Wilms group	1,082 (966–1,082)	–
Neuroblastoma group	627 (57–2,364)	0.082
**HVA (mg/24 h)**		
Study group	58.8 (29.4–91.6)	
Wilms group	–	–
Neuroblastoma group	239 (18–1,269)	0.086
**VMA (mg/24 h)**		
Study group	29.2 (4.8–71.5)	
Wilms group	–	–
Neuroblastoma group	82.3 (6.8–231)	0.073

The ultrasound study analyzed heterogeneity, lobulated outline, size, tumor restricted to the kidney, and Doppler flowmetry. No data was statistically significant, but the mean size of the study group was half that of the WT group (6.5 vs. 12.7, *p* = 0.155) and similar to that of the neuroblastoma group (6.5 vs. 7.1, *p* = 0.447) (Table 3). The parameters analyzed at CT were heterogeneity, calcification, lymph node enlargement, capsular involvement, size, necrosis, crossing of the midline, and displacement of the great vessels. The only statistically significant result was calcification, which was present in 67% in the study group and absent in the WT group (*p* = 0.011). In the neuroblastoma group, 80% were found to have calcification (*p* = 0.550) (Table 4).

**Table 3:** Comparison between the study group, the Wilms group, and the neuroblastoma group in relation to US parameters.

**US parameters**		***p-*** **value**
**Heterogeneity**		
Study group	3	
Wilms group	6	0.696
Neuroblastoma group	4	0.696
US parameters		*p-*value
**Heterogeneity**		
Study group	3	
Wilms group	6	0.696
Neuroblastoma group	4	0.696
**Lobed outline**		
Study group	1	
Wilms group	1	0.696
Neuroblastoma group	1	0.696
**Size (cm)**		
Study group	6.5	
Wilms group	12.7	0.155
Neuroblastoma group	7.1	0.447
**Kidney restricted**		
Study group	4	
Wilms group	6	0.789
Neuroblastoma group	10	0.109
**Doppler flowmetry**		
Study group	1	
Wilms group	1	0.696
Neuroblastoma group	1	0.696
**Left side**		
Study group	4	
Wilms group	7	0.889
Neuroblastoma group	5	0.515
**Lobed outline**		
Study group	1	
Wilms group	1	0.696
Neuroblastoma group	1	0.696
**Size (cm)**		
Study group	6.5	
Wilms group	12.7	0.155
Neuroblastoma group	7.1	0.447
**Kidney restricted**		
Study group	4	
Wilms group	6	0.789
Neuroblastoma group	10	0.109
**Doppler flowmetry**		
Study group	1	
Wilms group	1	0.696
Neuroblastoma group	1	0.696
**Left side**		
Study group	4	
Wilms group	7	0.889
Neuroblastoma group	5	0.515

**Table 4:** Comparison between the study group, the Wilms group, and the neuroblastoma group in relation to CT parameters.

**CT parameters**		***p-*** **value**
**Heterogeneous**		
Study group	4	
Wilms group	8	0.55
Neuroblastoma group	7	0.889
**Calcification**		
Study group	4	
Wilms group	0	0.011^*^
Neuroblastoma group	8	0.55
**Lymph node enlargement**		
Study group	4	
Wilms group	4	0.301
Neuroblastoma group	3	0.152
**Capsular involvement**		
Study group	3	
Wilms group	3	0.423
Neuroblastoma group	1	0.073
**Size (cm)**		
Study group	11.3	
Wilms group	13.9	0.077
Neuroblastoma group	12.7	0.344
**Necrosis**		
Study group	3	
Wilms group	4	0.696
Neuroblastoma group	3	0.423
**Crosses midline**		
Study group	3	
Wilms group	2	0.21
Neuroblastoma group	0	0.385
**Displaces larges vessels**		
Study group	3	
Wilms group	3	0.423
Neuroblastoma group	2	0.21

* *Bold values means statistical significance (p < 0.05)*.

## Discussion

This is the first Brazilian study that focuses on the challenge of differential diagnosis between WT, neuroblastomas, and non-WT.

The study group had higher levels of pain, when compared to the WT controls (67% vs. 20%, *p* = 0.0611), and numbers very similar to the neuroblastoma controls, which can help to make differential diagnosis between WT and neuroblastoma, even though WTs can present with abdominal pain due to intratumoral bleeding or preoperative rupture.

Nowadays, there are various different image modalities to assist in the correct diagnosis of WT, but incorrect diagnosis still occurs in 5–12% of cases (14), which is a concern when the initial treatment is chemotherapy.

Despite having cases of misdiagnosis, diagnostic accuracy in the present series was 98.1%, which highlights the excellence of the multidisciplinary team and shows lower misdiagnosis rates than those described in the literature (15–18).

The diagnosis of WT tumor despite all the resources can be difficult in rare cases due to the intrarenal localization of neuroblastomas (4).

Even though complete resection is of essence in the treatment of solid tumors, accurate diagnosis is important due to the correct use of neoadjuvant chemotherapy (12).

While the abdominal mass or swelling is a sign almost always present in cases of WT, it is less common in neuroblastoma cases (19), which was also evidenced in our series.

Some CT findings, although not specific to a single type of tumor, may help the diagnosis. Displacement of large vessels, extension of the tumor beyond the midline, renal displacement, and calcifications are very suggestive of neuroblastomas but may be present in WT (20). In our series, tumor calcification was very characteristic of neuroblastoma, encompassing 80% of the cases in the study group and 80% of the cases in the neuroblastoma controls, whereas in the WT group, none of the cases had calcification. Table 5 shows that calcification was an important diagnostic finding for neuroblastoma both in our series and Dickson et al. A greater numbers of image parameters were analyzed in the present series compared to Dickson et al.

**Table 5:** Comparison between our data with those published by Dickson et al. (18).

**Authors**	**Carvalho et al. (2021)**	**Dickson et al. (18)**
Patients (*n*)	6	9
Final diagnosis	Neuroblastoma (5) Xanthogranulomatous pyelonephritis (1)	Neuroblastoma (9)
Patient age (months)	31.3 (11–47)	N/A
CT parameters	Heterogeneous (4) Calcification (4) Lymph node enlargement (4) Capsular involvement (3) Necrosis (3) Crosses midline (3) Displaces larges vessels (3)	Calcification (6) Vascular encasement (4)
HVA (mg/24 h)	58.8 (29.4–91.6)	86.5 (47–126)
VMA (mg/24 h)	29.2 (4.8–71.5)	300.8 (58–806)

In our study group, all patients had urinary catecholamines collected before surgery with the exception of the case of pyelonephritis. However, due to the delay in obtaining the results, it could not be used for treatment decision. Urinary catecholamine collection at presentation is very important and should be done with high priority to differentiate neuroblastoma cases.

Based on intraoperative findings, a trained surgeon sometimes suspects misdiagnosis and changes the surgical planning in order to achieve better prognosis without surgical morbidity. This can be illustrated in the two cases in which only biopsy was performed.

Further studies are needed to determine the occurrence of misdiagnosis in other Brazilian centers that use the SIOP protocol and evaluate the prognostic impact of preoperative treatment in tumors other than WT concerning survival.

## Conclusion

Some pathologies can be misdiagnosed as WT, especially when they present unspecified symptoms and dubious images. Diagnostic accuracy was 98.1%, which highlights the quality of the multidisciplinary team. The increase in abdominal volume is highly suggestive of WT, especially if associated with the absence of intratumoral calcifications on CT. If possible, urinary catecholamines should be collected before surgery as they help in the differential diagnosis of neuroblastoma.

## Data Availability Statement

The raw data supporting the conclusions of this article will be made available by the authors, without undue reservation.

## Ethics Statement

The studies involving human participants were reviewed and approved by Federal University of São Paulo—CEP #0808/13. Written informed consent to participate in this study was provided by the participants' legal guardian/next of kin.

## Author Contributions

LC: writing, data collection and data analysis. SA: study design, data review, and article final review. MA: data review and article review. HL: images analysis. MC: data review and article review. TK: writing, data collection and data analysis. All authors contributed to the article and approved the submitted version.

## Conflict of Interest

The authors declare that the research was conducted in the absence of any commercial or financial relationships that could be construed as a potential conflict of interest.

## Publisher's Note

All claims expressed in this article are solely those of the authors and do not necessarily represent those of their affiliated organizations, or those of the publisher, the editors and the reviewers. Any product that may be evaluated in this article, or claim that may be made by its manufacturer, is not guaranteed or endorsed by the publisher.
